# Prestressed Composite Polymer Gels as a Model of the Extracellular-Matrix of Cartilage

**DOI:** 10.3390/gels8110707

**Published:** 2022-11-02

**Authors:** Alexandros Chremos, Jack F. Douglas, Peter J. Basser, Ferenc Horkay

**Affiliations:** 1 Section on Quantitative Imaging and Tissue Sciences, Eunice Kennedy Shriver National Institute of Child Health and Human Development, National Institutes of Health, Bethesda, MD 20892, USA; 2 Materials Science and Engineering Division, National Institute of Standards and Technology, Gaithersburg, MD 20899, USA

**Keywords:** gels, extracellular matrix, cartilage

## Abstract

Articular cartilage is a composite hydrogel found in animal and human joints, which exhibits unique load-bearing properties that have been challenging to reproduce in synthetic materials and model in molecular dynamics (MD) simulations. We computationally investigate a composite hydrogel that mimics key functional properties of articular cartilage as a potential biomimetic model to investigate its unique load-bearing properties. Specifically, we find that the emergence of prestress in composite gels derives primarily from the stiffness of the polymer matrix and the asymmetry in the enthalpic interactions of the embedded particles and polymer matrix. Our MD simulations of the development of prestress agree qualitatively with osmotic pressure measurements observed in our model composite hydrogel material.

## 1. Introduction

Articular cartilage is a composite hydrogel that caps the ends of bones in mammalian joints and is responsible for transmitting loads through the body. Articular cartilage primarily consists of water, ions, and the extracellular matrix (ECM), which is the main component responsible for the cartilage’s mechanical properties. The ECM comprises a mesh of fibrous Type II collagen molecules, and aggrecan is a bottlebrush-shaped proteoglycan (PG) polymer entrapped within the collagen matrix forming complexes with hyaluronic acid (HA) [1]. The organization of the macromolecular components in the ECM depends on the tissue type and its biological function (e.g., it is different in cartilage and brain) [2,3,4,5]. One biological function of articular cartilage is to ensure proper lubrication of joints during repetitive physical activity, such as walking. Another basic function is to bear static and dynamic loads during activities such as jumping and standing. Thus, the ECM in articular cartilage must expel fluid under compressive loading in physical activity and recover its original shape and volume after unloading while minimizing joint wear [6,7]. Changes in the chemical composition and the organization of ECM’s macromolecular constituents reduces the load-bearing ability of cartilage, an effect associated with osteoarthritis (OA) [8,9,10,11]. Irreversible articular cartilage degradation can occur by injury (e.g., traumatic OA), aging, or inflammation (e.g., rheumatoid arthritis). Despite extensive research in characterizing articular cartilage’s properties, no viable synthetic or naturally-derived cartilage implants can replicate its remarkable biological functions.

An understanding of the nature of interactions between the components of articular cartilage can provide insights into its multiple biological functions and for the development of therapeutic treatments for diseases, such as osteoarthritis, in which cartilage loses its functional properties [7,12,13]. These insights should also be useful in understanding similar structurally complex gel-like tissues, such as the tendon, brain, and muscle. Synovial fluid located between the joints, as an example, is rich in HA acting as a ‘lubricant’ for the joints and as an elastic material when the joint movement is rapid or as a viscous liquid when the joint movement is slow [1]. Deviation from the physiological conditions of synovial fluid may cause implications for the cartilage’s ability to undergo reversible deformation, i.e., a hardened cartilage makes moving the surrounding joints more difficult and potentially causing pain. The lubricating and load-bearing ability is reduced in diseases such as osteoarthritis. This may occur as a result of a decrease in the available binding sites of the HA chain or as the result of damage to link proteins and the glycosaminoglycan chains. The size of PG aggregates within ECM decreases with age [14]. Aggregation may also affect pore size distribution and solute permeability [7]. However, the mechanisms that lead to the loss of cartilage’s key biomechanical properties remain poorly understood [1,7,12,13].

The creation of gels having mechanical properties (stiffness, etc.) similar to articular cartilage is a long-term scientific effort [15,16,17,18]. Poly(vinyl alcohol) (PVA) is typically used as a neutral polymer because it is relatively insensitive to changes in salt concentration, pH, or other changes in the composition of the surrounding solution. PVA gels, which are either chemically and/or physically cross-linked, are also widely used in biomedical applications [19,20,21]. However, the lack of viable synthetic cartilage implants not only necessitates the development of rational treatments to prevent joint damage, but also behooves us to obtain a deeper understanding of the physics and chemistry of cartilage-like gels to provide rational design principles to guide the development of functional cartilage implants. More generally, the development of such biomimetic cartilage-like materials and models, particularly through the use of polymer physics concepts, can provide a new fundamental understanding of the complex behaviors cartilage exhibits.

Horkay and Basser [22,23] recently proposed a novel composite hydrogel model of cartilage consisting of a poly(vinyl alcohol) (PVA) matrix, corresponding to the collagen matrix in articular cartilage, and cross-linked poly(acrylic acid) (PAA) microgel particles embedded within the PVA matrix, corresponding to the aggrecan/hyaluronic acid complex. The key feature of this composite hydrogel model is the development of stress that is present even in the absence of any external loading; we call this “prestress”. The existence of prestress in the cartilage plays a critical role in governing the tissue’s load-bearing ability. While this synthetic composite model exhibits a remarkable resemblance to articular cartilage in terms of load-bearing capabilities, a microscopic understanding based on polymer chemistry and the physics is required to explore the origin of the prestress. In addition to providing a road map to improve the performance of this composite hydrogel model in various applications (intervertebral disc spacers [24,25], shock absorbing materials [26], and sealants and caulks [27]), these compact gel particles entrapped in a network represent a new class of “squishy” materials whose study serves a larger scientific purpose while providing key insights into the normal function of cartilage and its dysfunction in abnormal development, disease, aging, and degeneration.

A similar strategy was used with the inclusion of cells in the extracellular matrix-mimicked tissue mechanics in weak interacting gel systems [28]. These authors pointed out that particle inclusions can modify the elastic response of gel-like materials, which has important implications on tissue stiffness. They also concluded that understanding the viscoelastic behavior of composite materials may shed light on the mechanisms by which tissue stiffness is altered in disease and determine how changes in the mechanical microenvironment lead to cellular dysfunction. This finding is also important in the design of new biomaterials with physiologically relevant mechanical properties.

To gain a deeper understanding of the various physical and chemical molecular and macromolecular-scale processes governing the overall macroscopic functional properties of this material, we develop a minimal composite gel model to investigate the conditions and molecular parameters that govern the material behavior in aqueous solvent environments. To gain insights into the two primary components, the network and the embedded particles, we developed models and methods to generate a wide range of gel-like structures ranging from compact [29] to open/fractal gel structures [30]. These models can also help us understand the importance of attractions in the degree of swelling and osmotic properties [31]. In the current study, we describe a composite gel model composed of a polymer matrix containing embedded self-associating branched particles. Using this model, we investigated the conditions by which prestress reinforces this type of gel under isotropic compression.

This paper is organized as follows: In Section 2, we discuss the investigation of the influence of the extracellular matrix’s topology and self-association of the embedded particles in the prestress of the composite gel. Our conclusions are presented in Section 3. Finally, Section 4 contains the details of the simulation methods and model, and the experimental methodology is presented in Section 4.2.

## 2. Results

At equilibrium, a swollen composite hydrogel coexists with the solvent by balancing two opposing forces. On the one hand, the gel swells due to the osmotic pressure of the polymer within the gel, $\Pi_{\mathrm{mix}}$. In the absence of cross-links, the polymer chains are completely dissolved (in a good solvent) due to thermodynamic interactions between the polymer and solvent molecules. On the other hand, in the presence of cross-links, the osmotic pressure of the polymer is counterbalanced by the elastic pressure, $\Pi_{\mathrm{el}}$, generated by the cross-links. Thus, we can formally decompose the osmotic pressure of the composite hydrogel, $\Pi_{\mathrm{com}}$, into two components,

(1) $$\Pi_{\mathrm{com}}=\Pi_{\mathrm{mix}}+\Pi_{\mathrm{el}}.$$

At equilibrium, we have the stability condition, $\Pi_{\mathrm{com}}=0$. In regular polymer gels, the elastic pressure, $\Pi_{\mathrm{el}}$, decreases with an increasing degree of swelling, reflecting the cross-link density decrease. The “prestress” $P_{\mathrm{el}}$ of the composite gel is defined as the difference of the pressure of the embedded particles ($\Pi_{\mathrm{emb}}$) and the osmotic pressure of the composite gel,

(2) $$P_{\mathrm{el}}=\Pi_{\mathrm{emb}}-\Pi_{\mathrm{com}}.$$

Based on this definition, it is clear that at equilibrium $P_{\mathrm{el}}$ will be $P_{\mathrm{el},\mathrm{eq}}\approx \Pi_{\mathrm{emb}}$. Thus, $P_{\mathrm{el},\mathrm{eq}}$ is dominated by the pressure exerted by the embedded particles. For small, relatively simple embedded particles, the osmotic pressure will be well described by a virial equation of the form of $M_{w,\mathrm{emb}}\Pi_{\mathrm{emb}}/\rho =1+B_{2}\rho +B_{3}\rho^{2}+\ldots$, where $B_{2}$ and $B_{3}$ are the second and third osmotic virial coefficients. The osmotic pressure contributed by the polymer matrix can be estimated by the Flory–Huggins expression [32],

(3) $$\Pi_{\mathrm{mix}}=-RT/V_{1}\left[\ln\left(1-\phi \right)+\phi +\chi_{0}\phi^{2}+\chi_{1}\phi^{3}\right],$$

where $V_{1}$ is the partial volume of the solvent, ϕ is the volume fraction of the polymer, $\chi_{0}$ and $\chi_{1}$ are interaction parameters related to the second and third virial coefficients, and *R* is the universal gas constant. The elastic contribution of the osmotic pressure is based on an expression that includes an account of intermolecular interactions in the gel, as discussed at length in Refs. [22,23],

(4) $$\Pi_{\mathrm{el}}=K_{1}\phi^{1/3}+K_{2}\phi^{4/3},$$

where $K_{1}$ and $K_{2}$ are fitting parameters. We also note that the Flory–Huggins expression for describing the osmotic pressure of semi-dilute polymers and gels is only applicable for neutral systems. However, previous experimental studies and molecular dynamics simulations have indicated that the Flory–Huggins formalism is satisfactory for phenomenologically describing polyelectrolytes in the presence of added salt at concentrations relevant to physiological relevance and at high polymer concentrations where the mean-field theory should apply [33,34,35].

**Figure 1:**
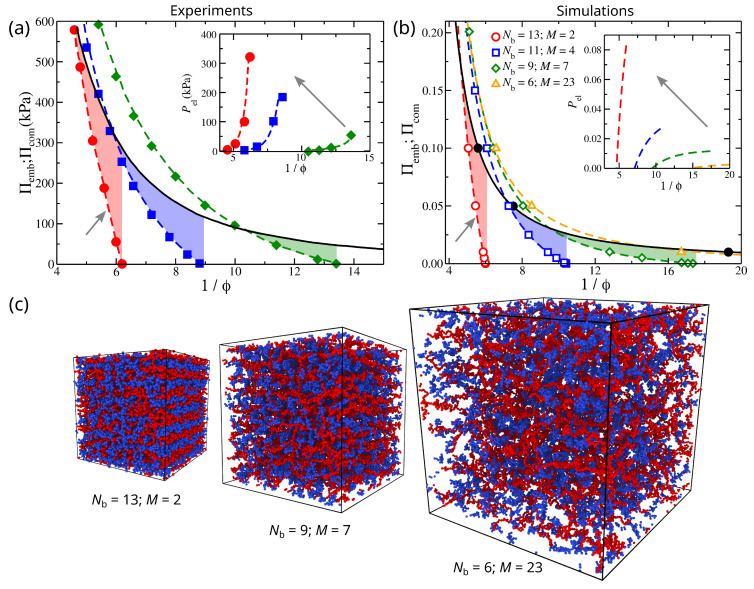
Osmotic pressure of composite gels, $\Pi_{\mathrm{com}}$, in: (**a**) experimental PVA/PAA varying network stiffness by changing the cross-linking density (dashed curves); and (**b**) simulation composite gel model having the strength of the attractions between them $\lambda_{\mathrm{emb}}=0.4$, as a function of the inverse of polymer fraction, 1/ϕ. In both (**a**,**b**), the osmotic pressure of embedded particles $\Pi_{\mathrm{emb}}$ is represented by the black continuous line, and in simulation results are represented by black filled circles. The highlighted regions outline the region at which the prestress $P_{\mathrm{el}}$ is calculated. The small arrows point to the composite gel that exhibits osmotic pressure similar to articular cartilage under physiological conditions [22]. Insets: $P_{\mathrm{el}}$ as a function of 1/ϕ. The gray arrows at the insets of (**a**,**b**) point in the direction of increasing the polymer network stiffness; (**c**) typical screenshots of composite gels having different gel architecture at Π=0.001 and $\lambda_{\mathrm{emb}}=0.4$.

The experimental composite gel model is a composite hydrogel composed of a poly(vinyl alcohol) (PVA) matrix that encapsulates weakly cross-linked poly(acrylic acid) (PAA) microgel particles. In Figure 1a, we find that the experimental osmotic pressure is different between a solution of embedded particles and the composite gel. As discussed in previous studies [22,23], the osmotic pressure difference between these two systems is due to the gel’s cross-linking and the influence of the filler on the composite gel’s osmotic pressure. An increase in the gel’s degree of cross-linking shifts the osmotic pressure curves to smaller values of 1/ϕ and increases the stiffness and the prestress of the composite gel, see inset of Figure 1a.

Our model is in good qualitative agreement with experimental observations [22], see Figure 1. We observe the same trends on how the mesh size of the polymer matrix influences the prestress of the composite gel, compare Figure 1a,b. We note that an increase in cross-linking effectively reduces the mesh size in a gel. In particular, for polymer matrices having the same total number of polymer matrix segments, then a decrease in the mesh size results in an increasing number of branched points in each direction while decreasing the chain length at the same time. In other words, we increase the density of branching points in the gel structure by decreasing the mesh size. We find that decreasing the mesh size results in a decrease in the capacity of the polymer matrix to swell, which, in turn, decreases the value of 1/ϕ at $\Pi_{\mathrm{com}}=0$, Figure 1b. A smaller chain length means that the polymer matrix becomes stiffer by resisting volumetric changes, resulting in a rapid increase of $\Pi_{\mathrm{com}}$ with ϕ. A smaller mesh size also means the formation of smaller compartments in the polymer matrix for the embedded particles to occupy, which effectively creates frustration in the capacity of the embedded particles to associate with each other. Thus, the design of composite gels lies in the ability to control the distribution of contacts between the different species and the strength of their interactions. A key difference between our model and experimental observations is the concavity of the prestress curves, see insets of Figure 1a,b, suggesting an additional layer of complexity in the model (e.g., incorporation of charges, salt, explicit solvent, etc.) is required to capture this feature. Understanding the origin of this difference will be a focus of future work.

**Figure 2:**
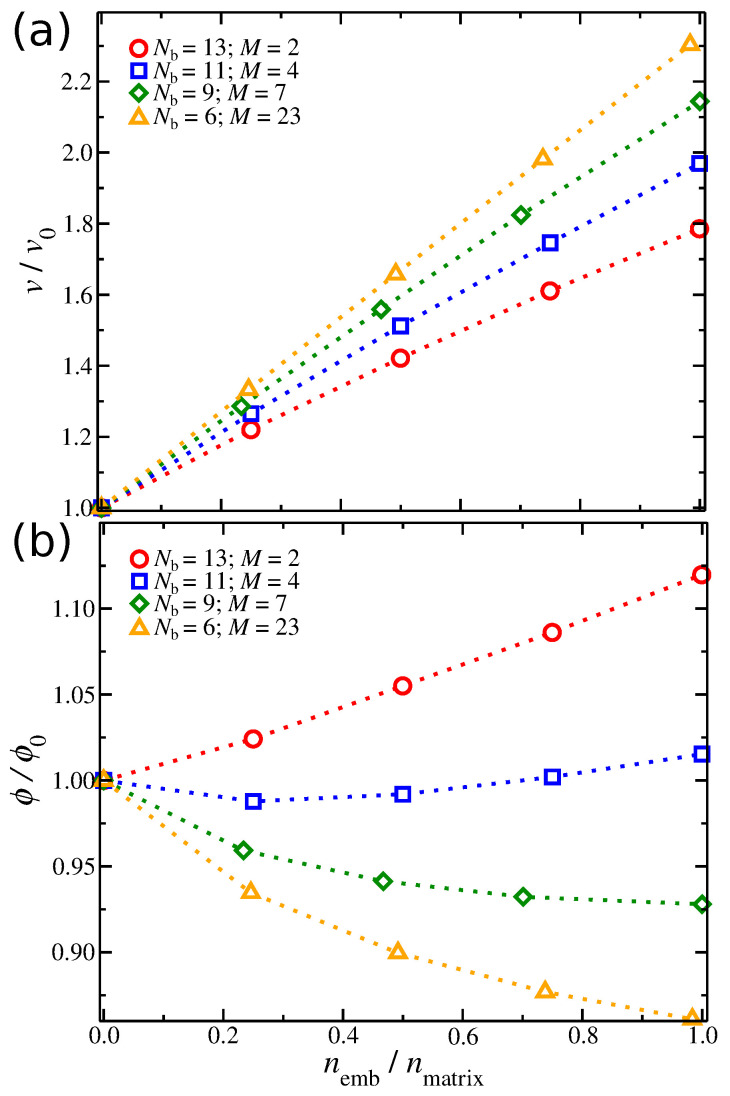
(**a**) Swelling of gel’s volume normalized by the volume of a gel without embedded particles, $v/v_{0}$, as a function of the segmental composition defined as the ratio of the total number of embedded particles’ segments over the total number of polymer matrix segments, $n_{\mathrm{emb}}/n_{\mathrm{mtx}}$, at fixed osmotic pressure of the composite gel, $\Pi_{\mathrm{com}}=0.01$. Results for different polymer matrices’ chain length *M* are also presented. (**b**) Volume fraction normalized by the volume fraction of a gel without embedded particles, $\phi /\phi_{0}$, as a function of $n_{\mathrm{emb}}/n_{\mathrm{mtx}}$, at $\Pi_{\mathrm{com}}=0.01$. For both (**a**,**b**), the strength of association of embedded particles was $\lambda_{\mathrm{emb}}=0.4$.

To understand the origin of the $P_{\mathrm{el}}$ in the composite gel, first, we need to examine the influence of the concentration of the embedded particles on the swelling of the extracellular matrix at fixed osmotic pressure conditions. In Figure 2a, we calculate the degree of the composite gel’s volume swelling normalized by the volume of the gel without the embedded particles. As expected, the volume of the composite gel increases progressively as more embedded particles are included in the gel. However, the polymer matrices exhibit a faster volumetric swelling rate by increasing the chain length. In other words, a shorter chain length in gels gives rise to a reduced capacity of the gel to swell. This swelling effect has a non-trivial dependence on ϕ. We calculated the ratio of ϕ over the volume fraction of a gel without embedded particles, Figure 2b. For M>4, the volume fraction of polymer matrix segments and embedded particles decreases as the number of embedded particles in the gel increases. That is because the gel structure swells faster than the embedded particles. For M=4, we find that $\phi /\phi_{0}$ remains at approximately $\phi /\phi_{0}\approx 1$, suggesting that at this chain length, the swelling rate is approximately the same with the increased number of embedded particles in the gel structure. For M=2, the ratio $\phi /\phi_{0}$ progressively increases as the number of embedded particles increases, resulting in an increased packing of embedded particles in the polymer matrix. The behavior of $\phi /\phi_{0}$ suggests to us a similarity to the Hofmeister series of electrolyte solutions [36,37], where the embedded particles play the role of salt. This hypothesis requires further study, however. At any rate, the reduced capacity of the polymer matrix to sufficiently swell to accommodate the insertion of embedded particles within its gel structure results in the build-up of prestress in the model composite gel.

**Figure 3:**
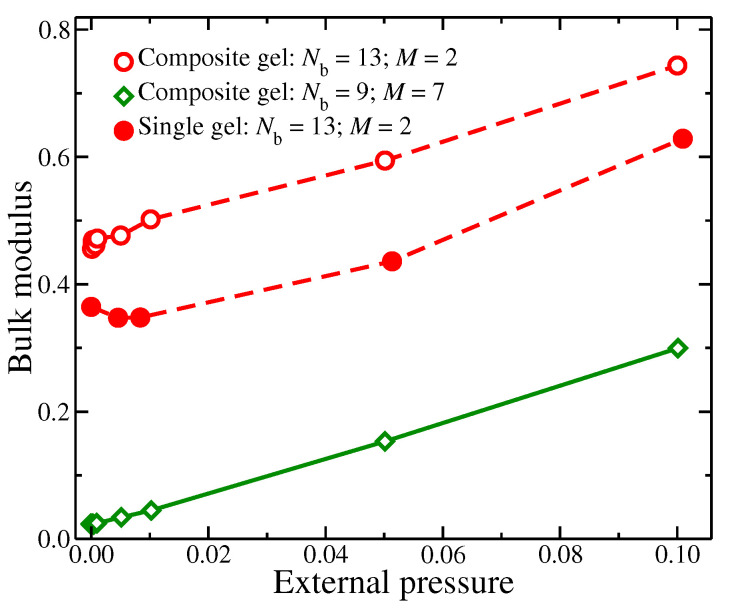
Bulk modulus, estimated as the inverse of isotropic compressibility $\kappa_{T}$, as a function of external pressure.

We also note that the molecular structure of the polymer matrix is just as important as the embedded particles. In Figure 3, we compare the bulk modulus ($\beta_{T}=1/\kappa_{T}$), where $\kappa_{T}$ is the inverse of the isotropic compressibility ($\kappa_{T}=-\frac{1}{V}\frac{\partial V}{\partial P}$), as a function of external pressure applied on the system. Bulk modulus provides a description of the material’s resistance to change volume. Evidently, the bulk modulus of the material increases significantly for higher density of branching points (defined as $\rho_{b}=N_{b}^{3}/v$, where *v* is the gel’s volume); we note that *v* is influenced by *M* as it determines the maximum extent of the polymer chains in the gel, as discussed above. For our model, we find that there is also a progressive increase of bulk modulus as the gels become more dense, which is perhaps unsurprising. We also find that the inclusion of embedded particles also increase the bulk modulus of the whole gel material, signifying that composite gels can be used towards the design of stronger gel materials.

**Figure 4:**
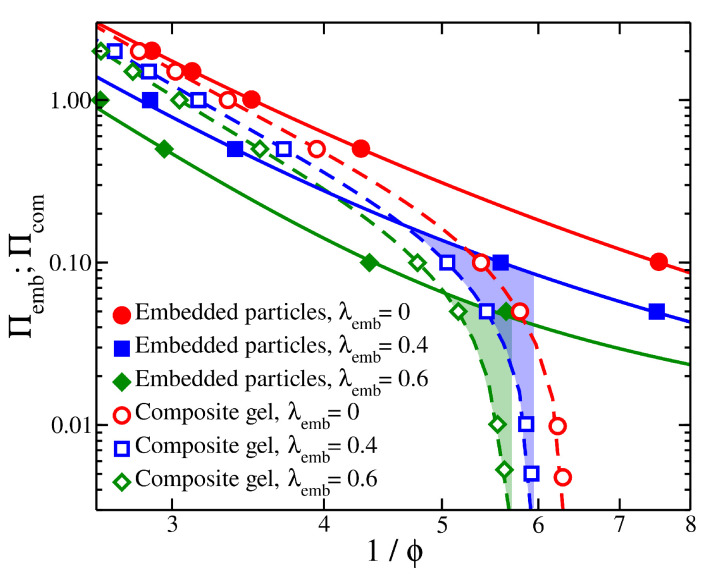
Osmotic pressure of composite gels $\Pi_{\mathrm{com}}$ (open symbols), with a polymer matrix having $N_{b}=13$ and chain length M=2, and osmotic pressure of embedded particles $\Pi_{\mathrm{emb}}$ (filled symbols) as a function of the inverse of polymer fraction 1/ϕ. The highlighted regimes outline the region at which the prestress is calculated. Results for different strengths of attractive interactions between the embedded particles are also presented.

**Figure 5:**
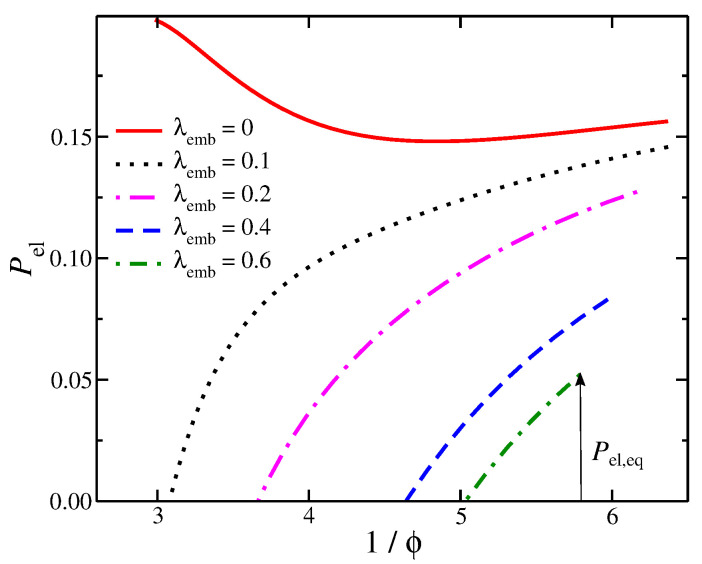
Prestress $P_{\mathrm{el}}$ of composite gels, with a polymer matrix having $N_{b}=13$ and chain length M=2, as a function of the inverse of the polymer fraction, 1/ϕ. Results at different strengths of attractive interactions between the embedded particles are also presented. The arrow defines the value of prestress at equilibrium, $P_{\mathrm{el},\mathrm{eq}}$, which occurs at $\Pi_{\mathrm{com}}=0$ for one of the systems.

The mechanical properties of the composite gel are significantly influenced by embedded particles. In our composite model, $P_{\mathrm{el}}$ progressively increases with 1/ϕ, which is a more subtle effect that leads to a maximum in $P_{\mathrm{el}}$; see the insets of Figure 1a,b. The cross-linking density of the polymer matrix can influence the prestress curves, as seen the insets of Figure 1a,b. An increase in the strength of self-association between the embedded particles, $\lambda_{\mathrm{emb}}$, decreases the height of osmotic pressure curves for both the composite gel and the embedded particle solution. However, an increase in $\lambda_{\mathrm{emb}}$ results in a more rapid decrease for $\Pi_{\mathrm{emb}}$, see Figure 4. This provides a mechanism for modulating the strength of attractive interactions between the embedded particles to control the value of $P_{\mathrm{el},\mathrm{eq}}$ and the range of $P_{\mathrm{el}}$. These observations illustrate the importance of self-association of the embedded particles through their dispersion interactions and the chemical cross-links of the polymer matrix in determining the $P_{\mathrm{el}}$ curves. Furthermore, the secondary structure formed by the self-association of the embedded particles (e.g., interparticle aggregates) influences the stiffness of the composite gel, as seen in Figure 3. $P_{\mathrm{el}}$ decreases as 1/ϕ decreases or alternatively reaches a plateau with 1/ϕ when all interactions are strongly repulsive, see Figure 5. Under these conditions, the behavior of $P_{\mathrm{el}}$ in our composite gel is basically the same as an ordinary gel. To avoid this behavior, we require $\Pi_{\mathrm{emb}}>\Pi_{\mathrm{com}}$ at low densities/pressures and $\Pi_{\mathrm{emb}}<\Pi_{\mathrm{com}}$ at high densities/pressures. This condition shows that there is a point at which $\Pi_{\mathrm{emb}}=\Pi_{\mathrm{com}}$, resulting in $P_{\mathrm{el}}=0$. At high densities, the pressure is dominated by the segment–segment interactions, which provide two potential approaches to making $\Pi_{\mathrm{emb}}<\Pi_{\mathrm{com}}$. The first approach is to increase the attractive interactions between the embedded particles. This type of interaction will decrease $\Pi_{\mathrm{emb}}$ and $\Pi_{\mathrm{com}}$, but its effect will be greater in $\Pi_{\mathrm{emb}}$ provided that the cross-interactions between the embedded particle’s segments and the segments of the polymer matrix remain fixed. This type of interaction effectively reduces the value of $B_{2}$ for the embedded particles. The second approach is based on increasing the strength of association between the embedded particles and the polymer matrix. This can be achieved by tuning non-dispersion type interactions, such as hydrogen bonding.

The influence of the attractive interactions between the embedded particles, regardless of whether these are self-interactions or cross-interactions, does *not* further inflate the polymer matrix. Indeed, the maximum inflation of the polymer matrix occurs when all the interactions are purely repulsive, at which $P_{\mathrm{el},\mathrm{eq}}$ is a minimum rather than a maximum. The attractive interactions enhance the association of the embedded particles, forming a loose network within the polymer matrix, which provides additional material rigidity under compression. We note that the attractions between the embedded particles need to be stronger than the attractions between the embedded particles and the polymer matrix to ensure that $\Pi_{\mathrm{emb}}<\Pi_{\mathrm{com}}$ for high pressures/densities. The results presented here are based on $\lambda_{\mathrm{cr}}=0$ (no cross-species attractions), but preliminary results (not shown here) support these observations. These lines of investigation will be part of future work.

The osmotic pressure of composite gels can be further tuned by varying the embedded particles’ molecular mass. At low particle densities, the osmotic pressure of the embedded particles is close to the ideal behavior, $\Pi_{\mathrm{emb}}/\rho \approx 1/M_{w,\mathrm{emb}}$. This suggests that at equilibrium $\Pi_{\mathrm{com}}\approx 0$ and according to Equation (2), $P_{\mathrm{el},\mathrm{eq}}$ increases by having embedded particles of smaller molecular mass, implying $P_{\mathrm{el},\mathrm{eq}}∼\rho /M_{w,\mathrm{emb}}$ at fixed density. The molecular mass and the strength of attractions of the embedded particles determine $P_{\mathrm{el},\mathrm{eq}}$. As the complexity of the model increases, such as by the inclusion of hydration, electrostatic, and pH effects, as well as entanglements, we anticipate that there will be more mechanisms available to tune the prestress behavior of these composite gels. The present study is a first step in the modeling of these complex gels and additional features such as charged monomers, hydration of the chain backbone and the monomers and counter-ions, etc,. so that we can address the apparent difference in the concavity between the prestress curves between experimental and simulation results. These extensions will require ongoing computational effort and additional measurements to validate the theory.

**Figure 6:**
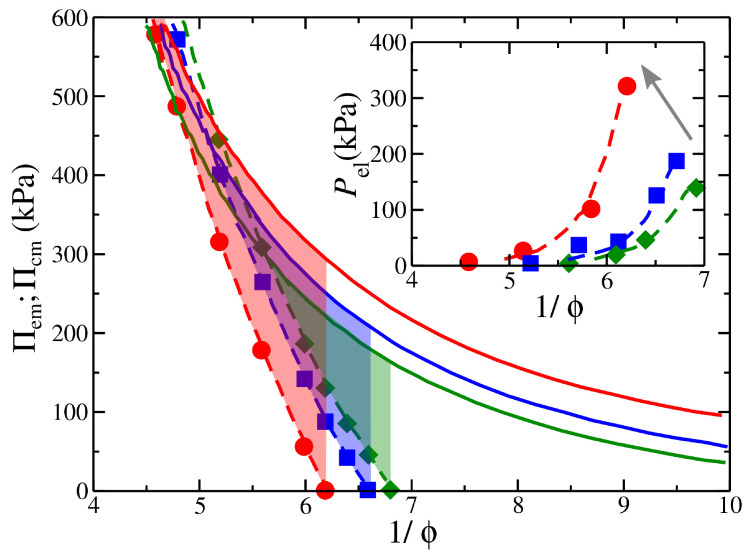
Experimental osmotic pressure of PVA/PAA composite gels containing PAA particles of varying nominal cross-link densities (dashed lines) and (continuous lines) PAA gels with varying cross-linking densities (high to low from left to right). The highlighted regions outline the region at which $P_{\mathrm{el}}$ is calculated. Inset: prestress $P_{\mathrm{el}}$ as a function of the inverse of polymer fraction, 1/ϕ. The gray arrow points in the direction of increasing the polymer network stiffness.

The optimal cross-link density of the embedded particles is not obvious. PAA embedded particles having low density of cross-links exhibit high swelling pressure that can eventually destabilize and destroy the PVA matrix. On the other hand, PAA particles having high density of cross-links may exhibit limited swelling that may not be sufficient to inflate the PVA gel. This also suggests that a composition should exist between these two extremes of low and high density cross-links where the composite gel exhibits the optimum mechanical properties. Increasing the cross-linking density of the embedded PAA gel particles also influences the prestress, see Figure 6. Increasing the cross-link density progressively reduces the swelling of the embedded particles and shifts the $\Pi_{\mathrm{com}}$ vs. 1/ϕ curves toward lower swelling pressures.

## 3. Conclusions

We propose a general model of composite gels that exhibits mechanical properties of high compressive load-bearing ability. This is achieved by creating a polymer matrix having a small mesh size and embedding in it self-associating small particles. A small mesh size polymer matrix has a large number of branched points that reinforce the polymer matrix, while the embedded particles inflate the volume of the polymer matrix. Combining these features results in new high-strength composite gel materials. In our framework, it becomes possible to tune the molecular parameters of the two components to create composite gels that mimic the biological function of articular cartilage [22,23]. Overall, we establish a minimal composite gel model that reproduces the behavior of high-strength composite gel materials for many potential applications. Our findings provide scientific insight into the molecular origin of the interactions between and among the different compartments and their role in determining key material properties in this class of materials.

## 4. Materials and Methods

### 4.1. Molecular Dynamics Simulation Methods and Models

We use a bead-spring polymer model suspended in an implicit solvent to describe a composite gel that exhibits mechanical properties similar to articular cartilage. All the segments, polymer matrix and embedded particles, have the same mass *m*, size σ, and strength of interaction ε; we assign ε and σ the units of energy and length, respectively.

The non-bonded interactions are described by an expression containing three terms [38,39].

First is a Lennard–Jones potential cut and shifted at the position of the minimum, $r_{\min}=2^{1/6}\sigma$, to describe the purely repulsive interactions, also know as the Weeks–Chandler–Andersen (WCA) potential [40],

(5) $$V_{\mathrm{WCA}}\left(r\right)=\left\{\begin{array}{cc} 4\varepsilon \left[{\left(\frac{\sigma }{r}\right)}^{12}-{\left(\frac{\sigma }{r}\right)}^{6}\right]+\varepsilon \; & r\leq r_{\min}\\ 0\; & r>r_{\min}. \end{array}\right.$$

The second term is a square-well (SW) potential representing the attractive interactions,

(6) $$V_{\mathrm{SW}}\left(r\right)=\left\{\begin{array}{cc} -\lambda \varepsilon \; & 0<r\leq r_{\min}\\ 0\; & r>r_{\min}, \end{array}\right.$$

where λ parameter provides a way to tune the effective monomer–monomer attractive interaction strength or the “solvent quality”. To ensure that the potential function does exhibit a discontinuity at $r=r_{\min}$ and smoothly reach V(r)=0 at a cut-off distance $r_{\mathrm{cut}}>r_{\min}$, we add the term,

(7) $$V_{\cos}\left(r\right)=\left\{\begin{array}{cc} \frac{1}{2}\lambda \varepsilon \left[\cos\left(\alpha r^{2}+\beta \right)-1\right]\; & r_{\min}<r\leq r_{\mathrm{cut}}\\ 0\; & r>r_{\mathrm{cut}}. \end{array}\right.$$

The parameters α and β satisfy the conditions $\alpha r_{\min}^{2}+\beta =\pi$ and $\alpha r_{\mathrm{cut}}^{2}+\beta =2\pi$. The cosine form of the potential also means that $dV_{\cos}/dr=0$ at $r=r_{\mathrm{cut}}$. We choose $r_{\mathrm{cut}}=3\sigma /2$, for which α and β become, $\alpha =\frac{4\pi }{9-4\sqrt[{3}]{2}}$ and $\beta =2\pi -\frac{9}{4}\alpha$.

**Figure 7:**
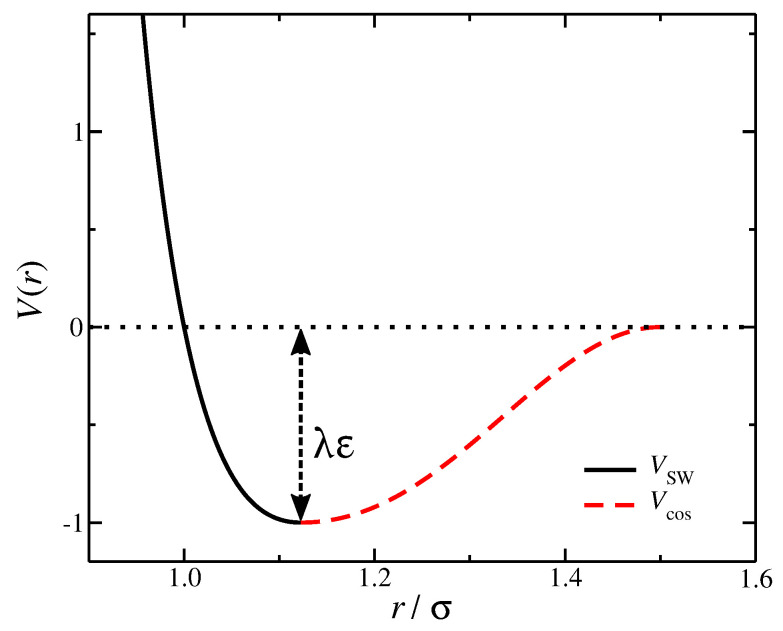
The non-bonded, segment–segment interaction potential V(r) at solvent-quality parameter λ=1. The different terms of V(r) are also presented, $V_{\mathrm{WCA}}\left(r\right)+V_{\mathrm{SW}}\left(r\right)$ (black) and $V_{\cos}\left(r\right)$ (red).

The non-bonded potential’s final form is $V\left(r\right)=V_{\mathrm{WCA}}+V_{\mathrm{SW}}\left(r\right)+V_{\cos}\left(r\right)$, see Figure 7. The solvent quality can be conveniently tuned by the parameter λ. In a good solvent, the entropic excluded volume interactions dominate over the enthalpic segment–segment interactions, which corresponds in effective purely repulsive segment–segment interactions with λ=0 in our model. In a poor solvent, the enthalpic dominates over the entropic excluded volume interactions, and this behavior can be modeled by attractive interactions with λ=1. This approach has been used frequently in the past in coarse-grained modeling of neutral polymers both in solution and in the melt [38,41,42]. The θ-solvent conditions for linear chains occur when λ=0.646 [38].

The segments along a chain are connected with their neighbors via a stiff harmonic spring, $V_{H}\left(r\right)=k{\left(r-l_{0}\right)}^{2}$, where $l_{0}=0.99\;\sigma$ is the equilibrium length of the spring, and $k=1000\;\varepsilon /\sigma^{2}$ is the spring constant.

The polymer matrix is composed of star polymer units placed in a cubic lattice with their free ends bonded with the free ends of the neighboring stars. This model is discussed at length in recent publications [29,30,31], except that in our study bonds through periodic boundary conditions are permitted. We label as $N_{x}$, $N_{y}$, and $N_{z}$ the number of branched points (or star polymers) in each direction [29]. Different molecular/lattice topologies can be used, but these are outside the scope of the current study. The molecular architecture of a regular star polymer is composed of a core particle and $f_{\mathrm{mtx}}$ linear chains each having *M* segments. One of the free ends of the linear chains is connected with the core particle. Thus, the molecular mass of the polymer matrix is $M_{w}=\left(N_{x}N_{y}N_{z}\right)\left(f_{\mathrm{mtx}}M+1\right)$. The polymer matrices in our study correspond to $N_{b}=N_{x}=N_{y}=N_{z}$ and $f_{\mathrm{mtx}}=4$ arms. The polymer matrix segment–segment interactions are purely repulsive with $\lambda_{\mathrm{mtx}}=0$.

**Figure 8:**
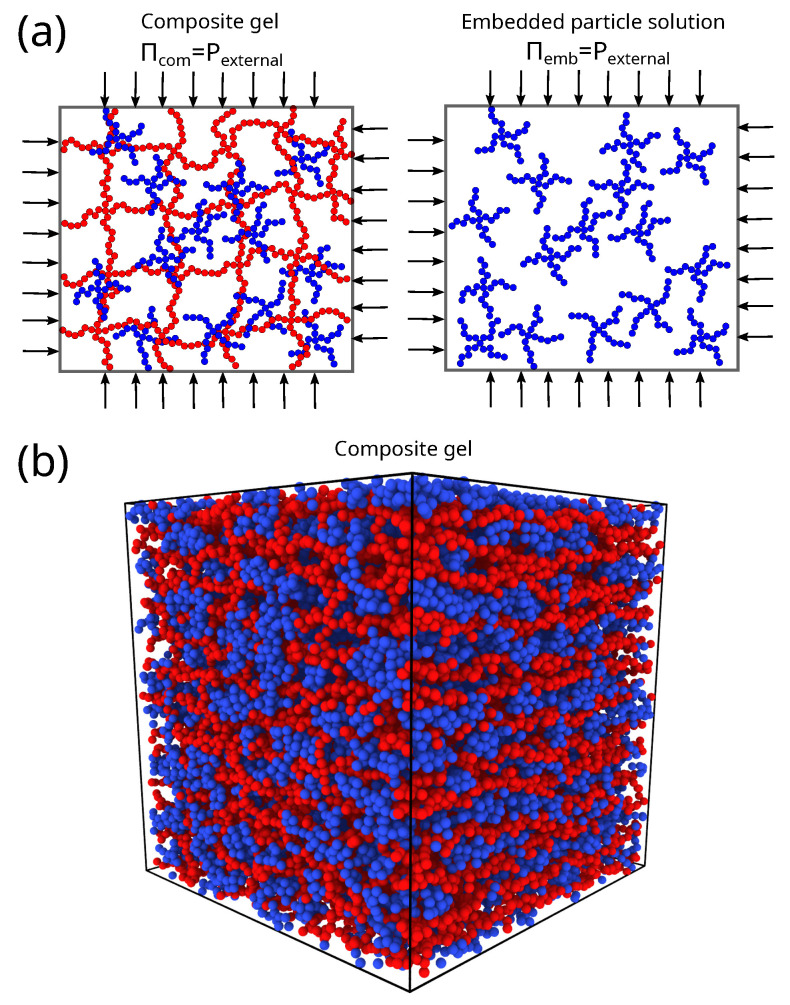
(**a**) Schematic of the composite gel model and embedded particle solution, where the polymer matrix is in red, and the embedded gel particles are in blue. These systems are at equilibrium by having the osmotic pressure equal to external pressure. (**b**) A screenshot of a composite gel model at equilibrium.

In the current study, the embedded particles are represented by a star polymer having $f_{\mathrm{emb}}=4$ arms and arm length $M_{\mathrm{emb}}=3$; the short length of the arms render this molecular structure to be highly spherically symmetric. The molecular mass of an embedded particle is $M_{w,\mathrm{emb}}=f_{\mathrm{emb}}M_{\mathrm{emb}}+1$. The embedded particles are inserted within the polymer matrix without overlaps, see Figure 8. The segment–segment interactions between the embedded particles is set by $\lambda_{\mathrm{emb}}$. The segment–segment cross-interactions between the embedded particles and polymer matrix are set by $\lambda_{\mathrm{cr}}$ and unless stated otherwise $\lambda_{\mathrm{cr}}=0$ for this study. The total number of embedded particles in the composite gel is such that the total number of segments of embedded particles, $n_{\mathrm{emb}}=N_{\mathrm{emb}}\left(f_{\mathrm{emb}}M_{\mathrm{emb}}+1\right)$, is approximately equal to the number of segments composing the polymer matrix, $n_{\mathrm{mtx}}=N_{b}^{3}\left(f_{\mathrm{mtx}}M_{\mathrm{mtx}}+1\right)$, unless stated otherwise. In other words, we primarily focus on composite gels having a composition $n_{\mathrm{emb}}/n_{\mathrm{mtx}}=1$. The volume fraction of the system is defined as, $\phi =\left(n_{\mathrm{emb}}+n_{\mathrm{mtx}}\right)v_{\mathrm{sphere}}/\langle v\rangle$, where 〈v〉 is the average volume of the system, and $v_{\mathrm{sphere}}$ is the volume of a sphere having a diameter σ, $v_{\mathrm{sphere}}=\frac{\pi }{6}\sigma^{3}$.

The systems were equilibrated by a Nosé–Hoover thermostat and barostat at constant temperature $k_{B}T/\varepsilon =1.0$ and constant pressure. The simulations were equilibrated for 5000τ, and data were accumulated over a 150,000τ interval, where $\tau =\sigma {\left(m/\varepsilon \right)}^{1/2}$ is the MD time unit; the time step used was Δt/τ=0.005.

The osmotic pressure of the system is determined using the virial equation $\Pi_{k}=\rho kT+W/V$, where the index *k* corresponds to the different types of gel systems, and the internal virial *W* is calculated from the sum of a pairwise virial function $w\left(r_{ij}\right)=r_{ij}dU\left(r_{ij}\right)/dr_{ij}$.

### 4.2. Preparation and Characterization of Composite PVA/PAA Hydrogels

In the present study, we synthesized composite hydrogels using the procedure we have previously proposed [22]. We combine chemical cross-linking of PVA with partial crystallization induced by freeze–thawing. PVA/PAA composite gels were prepared by dispersing PAA microgel particles in a PVA solution (1:1 ratio) prior to PVA cross-linking. The size of the PAA particles was in the range of 5 μm to 10 μm. Glutaraldehyde was used to cross-link the PVA molecules. The gels were equilibrated with 100 mol/m${}^{3}$ NaCl solution. The swelling pressure of the gels $\Pi_{\mathrm{com}}$ was determined by an osmotic stress technique [43]. In this method, gels enclosed in a dialysis bag are equilibrated with polymer solutions of known osmotic pressure [44]. The concentrations of the gels and the polymer solutions are determined at equilibrium. This procedure yields the dependence of $\Pi_{\mathrm{com}}$ on the polymer volume fraction ϕ.

## Data Availability

Available upon request.
